# Supplementary material for: Influence of sand content on mechanical properties of sand-silt mixtures from check dam deposits in the Loess Hilly of Ningxia, China
Source: PLoS One. 2023 Nov 2;18(11):e0287552. doi: 10.1371/journal.pone.0287552 (PMC10621870; doi:10.1371/journal.pone.0287552)
Supplement: Attachment — Submitted filename: Response to Reviewers.docx [file pone.0287552.s002.docx]

Modification  Explanation

Dear Editors and Reviewers:

Thank you for your letter and for the reviewers' comments concerning our manuscript entitled "**Influence of sand content on the mechanical properties of sand-silt mixtures from check dam deposits in the Loess Hilly of Ningxia, China"** (PONE-D-23-02538). Those comments are all valuable and very helpful for revising and improving our paper, as well as the important guiding significance to our research. We have studied the comments carefully and have made corrections which we hope meet with approval. Revised portions are marked in red in the paper. The main corrections in the paper and the responses to the reviewer's comments are as follows:

Responds to the reviewer's comments:

**Reviewer #1:**

**1. Abstract is not written appropriately. More precise results should be summarized.**

**Response:** Considering the reviewer's suggestion, we have rewritten the Abstract, and the important conclusions of the paper are summarized in the Abstract.

**2. Novelty and significance of this study should be addressed.**

**Response:** Considering the reviewer's suggestion, the novelty and significance of the study have been addressed in the Research Significance.

**3. Comparing fitted results to the measured ones is not adequate.**

**Response:** Considering the reviewer's suggestion, Adding two new cases is to verify the reliability of the Modified Duncan-Chang model proposed in this paper.

**4. The literature review lacks thorough understanding on the recent developments in this research field. ground improvement methods should be explained.**

**Response:** Thank you for the references provided by the reviewers. Some research about the engineering characteristics of sand-fine mixtures in the past three years has been added to the literature review.

**5. The paper reads like a short report. The authors must improve the Discussion in the paper. Avoid general conclusions and mention limitations of their work.**

**Response:** Considering the reviewer's suggestion, we have supplemented the discussion section in the paper, and the limitations of our work are mentioned in the Discussion. The main conclusions of the paper have been summarized in the Conclusion.

**6. Validity and repeatability of the experiments and results should be checked.**

**Response:** Considering the reviewer's suggestion, the validity and repeatability of the experiments and results have been checked.

**7. A detailed experimental procedure and the accuracy of different components of the experimental system are given. Measurement equipments should be described. And also, the characteristics of measurement equipments should be given.**

**Response:** Considering the reviewer's suggestion, the detailed experimental procedure and the accuracy of different components of the experimental system have been described in the Experimental Equipment and Experimental Procedures, respectively. The characteristics of measurement equipment have also been described in the Experimental Equipment.

Special thanks to you for your good comments.

**Reviewer #2:**

**1. The paper studied the influence of sand content on the mechanical properties of sand-silt mixtures from check dam deposits in the Loess Hilly of Ningxia, China, in which the CD tests were performed and the Duncan-Chang model was revised to describe the stress-strain relationship. The volumetric strain is not provided there, and the samples behave strain softening, which can not describe the Duncan-chang model. In view of this, the paper can not be accepted for publication.**

**Response:** It is really true, as the reviewer suggested that the volumetric strain is not provided in the paper. Considering the reviewer's suggestion, we have supplemented this part of the experimental data in the paper. As the reviewer said, the initial Duncan-Chang model can not characterize the softening stress-strain curves of geomaterials. However, the results of the triaxial test in the study show that the stress-strain relationships of sand-fine mixtures with different sand contents under various confining pressures have the characteristic of strain softening. In order to apply the Duncan-Chang model to the quantitative analysis of the effect of sand content on the mechanical properties of sand-fine mixtures, the model must be further modified to reflect the strain-softening behavior of the soil. Then a modified Duncan-Chang model is developed based on the initial Duncan-Chang model, and its reliability in describing the softening stress-strain curves is verified by comparing the predicted stress-strain curves with the measured curves of coral clay and undisturbed loess. Finally, a sand-content-dependent constitutive model that considered the effects of sand content and confining pressure of the soil by constructing the relationship between model parameters and confining pressure and sand content is proposed based on the modified Duncan-Chang model. The constitutive model is implemented in ABAQUS (version 6.14) software and verified by comparing the calculated results with the triaxial test data of sand-fine mixtures under the confining pressure of 500 kPa. The comparison results indicate that the constitutive model can reflect the real characteristics of sand-fine mixtures.

Special thanks to you for your good comments.

**Journal requirements:**

**1. Thank you for stating the following financial disclosure:**

**"The study was supported by the Natural Science Foundation of China (Project No.41962016) and the Doctoral Scientific Fund Project of the Ministry of Education of China (Project No.20136401110003)"**

**Please state what role the funders took in the study. If the funders had no role, please state: "The funders had no role in study design, data collection and analysis, decision to publish, or preparation of the manuscript."**

**If this statement is not correct you must amend it as needed.**

**Please include this amended Role of Funder statement in your cover letter; we will change the online submission form on your behalf.**

**Response:** The Natural Science Foundation of China (Project No.41962016) and the Doctoral Scientific Fund Project of the Ministry of Education of China (Project No.20136401110003) play an important role in the study design, data collection and analysis, and preparation of the manuscript, and the amended role of these funders have been stated in the cover letter.

**2. Please include a copy of Table 4 which you refer to in your text on page 12.**

**Response:** The specific content of Table 4 is as follows:

**Table 4. Values of *K* and *n* for different sand contents**

| $\omega_{s}$ | $\sigma_{3}$ | $\lg\left(\sigma_{3}/p_{a}\right)$ | $\lg\left(E_{0}/p_{a}\right)$ | *K* | *n* |
| --- | --- | --- | --- | --- | --- |
| /% | /kPa |  |  |  |  |
| 0 | 100 | 0.000 | 0.190 | 1.559 | 1.118 |
|  | 200 | 0.301 | 0.523 |  |  |
|  | 300 | 0.477 | 0.754 |  |  |
|  | 400 | 0.602 | 0.848 |  |  |
| 16.67 | 100 | 0.000 | 0.262 | 1.853 | 1.051 |
|  | 200 | 0.301 | 0.589 |  |  |
|  | 300 | 0.477 | 0.795 |  |  |
|  | 400 | 0.602 | 0.880 |  |  |
| 28.57 | 100 | 0.000 | 0.456 | 2.767 | 0.865 |
|  | 200 | 0.301 | 0.683 |  |  |
|  | 300 | 0.477 | 0.840 |  |  |
|  | 400 | 0.602 | 0.985 |  |  |
| 50 | 100 | 0.000 | 0.295 | 1.923 | 1.125 |
|  | 200 | 0.301 | 0.634 |  |  |
|  | 300 | 0.477 | 0.815 |  |  |
|  | 400 | 0.602 | 0.976 |  |  |
| 60 | 100 | 0.000 | 0.132 | 1.459 | 1.240 |
|  | 200 | 0.301 | 0.646 |  |  |
|  | 300 | 0.477 | 0.697 |  |  |
|  | 400 | 0.602 | 0.913 |  |  |

**3. In your Methods section, please provide additional information regarding the permits you obtained for the work. Please ensure you have included the full name of the authority that approved the field site access and, if no permits were required, a brief statement explaining why.**

**Response:** There are no permits which are required for the word. This is because all work is completed by myself and other research group members without involving third-party organizations.

Special thanks to you for your good comments.

We tried our best to improve the manuscript and made some changes in the manuscript. These changes, which are marked in red in the revised paper, will not influence the content and framework of the paper.

We appreciate for Editors/Reviewers' warm work earnestly and hope that the correction will meet with approval.

Once again, thank you very much for your comments and suggestions.

Hongyu Wang (Corresponding author)

Ningxia university

May 23, 2023
